# Supplementary material for: A Comprehensive Assessment of Ultraviolet-Radiation-Induced Mutations in Flammulina filiformis Using Whole-Genome Resequencing
Source: J Fungi (Basel). 2024 Mar 20;10(3):228. doi: 10.3390/jof10030228 (PMC10971301; doi:10.3390/jof10030228)
Supplement: Supplementary file 1 [file jof-10-00228-s001.zip › Supplementary Material S8/KEGG annotation/out/64381550635650.os/KO/out_map/map04911.html]

KEGG PATHWAY: Insulin secretion - Reference pathway


|  |  |
| --- | --- |
| **Insulin secretion - Reference pathway** |  |

[
Pathway menu
| Organism menu
| Pathway entry
| Show description
| User data mapping
]

Pancreatic beta cells are specialised endocrine cells that continuously sense the levels of blood sugar and other fuels and, in response, secrete insulin to maintain normal fuel homeostasis. Glucose-induced insulin secretion and its potentiation constitute the principal mechanism of insulin release. Glucose is transported by the glucose transporter (GLUT) into the pancreatic beta-cell. Metabolism of glucose generates ATP, which inhibits ATP-sensitive K+ channels and causes voltage-dependent Ca2+ influx. Elevation of [Ca2+]i triggers exocytotic release of insulin granules. Insulin secretion is further regulated by several hormones and neurotransmitters. Peptide hormones, such as glucagon-like peptide 1 (GLP-1), increase cAMP levels and thereby potentiate insulin secretion via the combined action of PKA and Epac2. Achetylcholine (ACh), a major parasympathetic neurotransmitter, binds to Gq-coupled receptors and activates phospholipase C- (PLC-), and the stimulatory effects involve activation of protein kinase C (PKC), which stimulates exocytosis. In addition, ACh mobilizes intracellular Ca2+ by activation of IP3 receptors.

|  |  |
| --- | --- |
| Reference pathway | 100% |
